# Genome-wide identification and expression analysis of the C2H2-zinc finger transcription factor gene family and screening of candidate genes involved in floral development in *Coptis teeta* Wall. (Ranunculaceae)

**DOI:** 10.3389/fgene.2024.1349673

**Published:** 2024-01-22

**Authors:** Shao-Feng Duan, Yan Zhao, Ji-Chen Yu, Gui-Sheng Xiang, Lin Xiao, Rui Cui, Qian-Qian Hu, Timothy Charles Baldwin, Ying-Chun Lu, Yan-Li Liang

**Affiliations:** ^1^ The Key Laboratory of Medicinal Plant Biology of Yunnan Province, National-Local Joint Engineering Research Center on Gemplasm Innovation and Utilization of Chinese Medicinal Materials in Southwest, College of Agronomy and Biotechnology, Yunnan Agricultural University, Kunming, Yunnan, China; ^2^ Yunnan Land and Resources Vocational College, Kunming, Yunnan, China; ^3^ Zhongshan Zhongzhi Pharmaceutical Group Co., Ltd., Zhongshan, Guangdong, China; ^4^ Faculty of Science and Engineering, University of Wolverhampton, Wolverhampton, United Kingdom; ^5^ Yunnan Agricultural University College of Education and Vocational Education, Yunnan Agricultural University, Kunming, Yunnan, China

**Keywords:** bioinformatics, C2H2-zinc finger transcription factors, genome-wide identification, *Coptis teeta* Wall, floral development

## Abstract

**Background:** C2H2-zinc finger transcription factors comprise one of the largest and most diverse gene superfamilies and are involved in the transcriptional regulation of flowering. Although a large number of C2H2 zinc-finger proteins (C2H2-ZFPs) have been well characterized in a number of model plant species, little is known about their expression and function in *Coptis teeta*. *C. teeta* displays two floral phenotypes (herkogamy phenotypes). It has been proposed that the C2H2-zinc finger transcription factor family may play a crucial role in the formation of floral development and herkogamy observed in *C. teeta*. As such, we performed a genome-wide analysis of the C2H2-ZFP gene family in *C. teeta*.

**Results:** The complexity and diversity of *C. teeta* C2H2 zinc finger proteins were established by evaluation of their physicochemical properties, phylogenetic relationships, exon-intron structure, and conserved motifs. Chromosome localization showed that 95 members of the C2H2 zinc-finger genes were unevenly distributed across the nine chromosomes of *C. teeta*, and that these genes were replicated in tandem and segmentally and had undergone purifying selection. Analysis of cis-acting regulatory elements revealed a possible involvement of C2H2 zinc-finger proteins in the regulation of phytohormones. Transcriptome data was then used to compare the expression levels of these genes during the growth and development of the two floral phenotypes (F-type and M-type). These data demonstrate that in groups A and B, the expression levels of 23 genes were higher in F-type flowers, while 15 genes showed higher expressions in M-type flowers. qRT-PCR analysis further revealed that the relative expression was highly consistent with the transcriptome data.

**Conclusion:** These data provide a solid basis for further in-depth studies of the C2H2 zinc finger transcription factor gene family in this species and provide preliminary information on which to base further research into the role of the C2H2 ZFPs gene family in floral development in *C. teeta*.

## Introduction

Floral development is key to plant reproductive biology. A large number of studies have shown that transcription factors (TF), play a crucial role in the complex transcriptional regulation of flower development (Moreau et al., 2016; Sharma et al., 2016; Prunet et al., 2017; Hugouvieux et al., 2018; Lyu et al., 2020). Among these transcription factors, C2H2-zinc finger proteins (C2H2-ZFPs) are one of the largest and most diverse superfamilies (Böhm et al., 1997). In *Arabidopsis thaliana*, the C2H2-ZFPs are divided into three sets, namely, A-set, B-set, and C-set. Zinc-finger domains (ZF) arrays are composed of tandem ZFs linked by up to ten amino acid residues, with five amino acid residues being the most frequent linker length (Englbrecht et al., 2004). The ZFPs in set A possess one ZF array and the ZFPs in set B, possess more than one array of tandem ZF. Both groups may present another’s dispersed ZFs besides the ZF arrays. The ZFPs in set C possess one or more dispersed ZFs.

The first C2H2-zinc finger gene was cloned in a study of *Petunia hybrida* petal development (Takatsuji et al., 1992), after which, the role of C2H2-ZFPs in flower development in other angiosperms was initiated. As a result of which, it has been shown that C2H2-ZFPs are involved in the transcriptional regulation of flowering induction, floral organ development, pollen and pistil development in all angiosperms studied to date (Payne et al., 2004; Borg et al., 2014; Li et al., 2016a; Lyu and Cao, 2018; Xu et al., 2018; Lyu et al., 2020). For example, in tomato (*Solanum lycopersicum*) it has been found that a single base mutation (C-T), of one gene (*SE3.1*) belonging to the C2H2-zinc finger transcription factor family, leads to a flat style being converted into a short style. Overexpression of this gene in tomatoes with flat styles resulted in a phenotype in which the stigma was exposed in immature flowers (Shang et al., 2021). In addition, previous studies have identified C2H2-ZFPs in some species, such as 176 C2H2-ZFPs in *Arabidopsis thaliana* (Englbrecht et al., 2004), 218 C2H2-ZFPs in *Medicago truncatula*, 109 C2H2-ZFPs in *Populus trichocarpa* (Liu et al., 2015), 97 C2H2-ZFPs in *Citrus sinensis* (Jia et al., 2023), 141 C2H2-ZFPs in *Brassica rapa* (Ma et al., 2022), 115 C2H2-ZFPs in *Panax ginseng* Meyer (Jiang et al., 2022), 457 C2H2-ZFPs in *Triticum aestivum* L. (Li et al., 2021), and 18 C2H2-ZFPs in *Pleurotus ostreatus* (Ding et al., 2022). However, to date no C2H2-ZFPs have been identified in *Coptis teeta.*

*C. teeta* is a perennial, alpine species indigenous to the low-latitude, alpine environment of southwest China, which belongs to the genus *Coptis* within the family Ranunculaceae (Liu et al., 2020). In our study, we identified two floral phenotypes to be present in the Yunnanese population of *C. teeta*; the first type possessed a long pistil with short stamens (F-type) and the second possessed a short pistil with long stamens (M-type). This indicated that this population displayed a typical herkogamy phenotype. As such, we believe that *C. teeta* may represent a useful model system, for the investigation of floral development in general and the processes of herkogamy in particular.

In order to gain an insight into the role of the C2H2-zinc finger transcription factor family in the formation of floral development and herkogamy in this species, a genome-wide experimental methodology was employed. One hundred C2H2-zinc finger genes from *C. teeta* were shown to be highly homologous to C2H2-zinc finger proteins in other plant species. The genetic and molecular characteristics of these genes, including phylogenetic relationships, gene structure, conserved motifs, exon-intron organization, and predictions of their chromosomal location were subsequently elucidated. Furthermore, in order to understand the potential function of these genes in floral development and organ formation, their patterns of expression. Related to flower organ identity during flower development were investigated and verified using real-time quantitative polymerase chain reactions (qRT-PCR). These data provided further molecular details of the spatial and temporal expression patterns of this gene family, which may assist the future elucidation of the molecular mechanism of floral organ development and herkogamy formation in this species.

## Materials and methods

### Plant material, DNA extraction and genome sequencing

Plant material was collected from the low-latitude, alpine environment of southwest China (E 95°48, N 25°09 to E 98°52, N 29°51). High-quality genomic DNA was prepared from fresh leaves of a single *C. teeta* plant, using a modified CTAB method (Porebski et al., 1997). This DNA was subsequently used for Illumina, PacBio, and Hi-C library construction. Genomic data was also acquired, but is yet to be published.

The plant materials used for total RNA extraction for the transcriptome sequencing and qRT-PCR were harvested at four stages of flower development: stage 1. The bracts were dehiscent and florets unexposed; stage 2: The florets were exposed, and the sepals were unexpanded; stage 3: The sepals had fully expanded; stage 4: The anthers had fully dehisced and begun to disperse pollen (Supplementary Figure S1).

### Identification and characterization of the C2H2-zinc finger genes in *C. teeta*

The hidden Markov model (HMM) of the C2H2-zinc finger protein domains was retrieved from the Pfam database (PF00096) (El-Gebali et al., 2019) and was used to extract the sequences containing conserved domains by TBtools (Chen et al., 2020). All the *C. teeta* C2H2-ZFPs sequences obtained were confirmed using the SMART program (http://smart.embl-heidelberg.de/) (Letunic et al., 2021) and the InterPro (http://www.ebi.ac.uk/interpro/search/sequence/) (Zdobnov and Apweiler, 2001). Sequences that did not contain C2H2-zinc finger domains were then removed and the remaining genes which encoded for proteins containing C2H2-ZF domains were used for further analysis. In addition, the ExPASy Server Tool (http://web.expasy.org/compute_pi/) (Gasteiger et al., 2005) and TBtools (Chen et al., 2020) was used to predict the physicochemical properties such as the theoretical isoelectric point (PI), instability index, aliphatic index, hydrophilic index, and molecular weight (MW) of the predicted proteins encoded by these genes. Finally, we made a prediction of their subcellular localization (Chou and Shen, 2010).

All ZFPs containing tandem ZFs domains in one array, or in more than one array, were assigned accordingly to A or B sets respectively, all ZFPs containing a single ZF or dispersed ZFs were assigned to the C set. Based on the results of our statistical analysis of linker lengths in the ZFPs, we defined tandem ZFs as fingers linked by up to ten amino acid residues, with five residues as the most frequent (consensus) linker length (Englbrecht et al., 2004; Arrey-Salas et al., 2021).

### Phylogenetic analysis of C2H2-zinc finger genes in *C. teeta*

The C2H2-zinc finger genes of *Arabidopsis thaliana* were retrieved from the TAIR website (https://www.arabidopsis.org/browse/genefamily/index.jsp) (Rhee et al., 2003). By using the C2H2-zinc finger protein sequences of *Arabidopsis thaliana* as templates, the C2H2-zinc finger protein sequences of *C. teeta* were identified using the multiple sequence alignment by employment of MAFFT v7.490 software. A phylogenetic tree was constructed using the maximum likelihood (ML) method, using IQ-TREE v2.2.0-beta software, the model was JTT + F + R5, and the parameter was set to-B 2000-alrt 2000 (Rozewicki et al., 2019).

### Identification and analysis of gene structure, promoter elements and conserved motifs

First, the genomic data of *C. teeta* was obtained and analyzed using TBtools software. Second, the 2000bp upstream sequence of coding sequence (CDS) was used for cis-regulatory element analysis via the PlantCare website (Lescot et al., 2002). The MEME online website (Bailey et al., 2009) was then used to identify conserved C2H2-zinc finger motifs. A MEME search was executed with the following parameters: any number of repetitions, the maximum number of motifs = 10. Finally, these results were then visualized with TBtools (Chen et al., 2020).

### Chromosomal locations and duplication analysis

*C. teeta* C2H2-zinc finger genes were mapped onto the chromosomes based upon the genomic data. The gene distribution map on the chromosomes of *C. teeta* was drawn by TBtools software (Chen et al., 2020). In addition, this software was used to estimate the non-synonymous substitution rate (Ka) and synonymous substitution rate (Ks).

### Expression analysis of *C. teeta* C2H2-zinc finger genes

Total RNA was extracted from fresh M and F type flowers using a kit, according to the manufacturer’s instructions (Magen, Guangzhou, China). The concentration of the extracted total RNA was measured using the NanoDrop technique (Thermo Fisher Scientific, United States), the equivalent amount RNA was reverse transcribed into cDNA using a reverse transcription kit (TAKARA, Beijing, China). Six candidate genes were chosen to verify the RNA-seq data. Quantitative real-time PCR (qRT-PCR) was performed using an Applied Biosystems QuantStudio 5 system (Thermo Fisher Scientific, United States) with the ChamQ Universal SYBR qPCR Master Mix. The PCR reaction was performed as follows: pre-denaturation 95°C for 30 s, denaturation at 95°C for 30 s, annealing at 58°C for 30 s, this reaction was repeated for 40 cycles. Subsequently, an extra procedure was conducted as follows: denaturation at 95°C for 15 s, annealing at 60°C for 1 min, extension at 72°C for 15 s. The fluorescence signal was then detected, and the dissolution curve analyzed. The primer sequences of the candidate genes are listed in Table 1. The relative expression patterns of the target genes were calculated using the 2^−ΔΔCT^ method and repeated in triplicate (Livak and Schmittgen, 2001). The selected expression patterns were visualized using TBtools (Chen et al., 2020).

**TABLE 1:** Primers of selected C2H2-zinc finger genes used in qRT-PCR experiments.

Gene name	Primer sequence (5′to3′)	Anneal temperature (°C)	GC content (%)	Length(bp)	Primer concentration(µM)
CteZFP28	5′F: TCA​ACA​AGT​GCG​AGA​AAA​CG	60.03	45	222	10
	3′R: ATT​TGA​GCT​CCC​ATT​GAT​GC	60.04	45		10
CteZFP43	5′F: TTC​TTC​ATG​GGA​CCC​ACT​TC	59.9	50	236	10
	3′R: CCG​GTG​TTG​GAA​GCA​GTA​GT	60.17	55		10
CteZFP95	5′F: ACC​AAC​ATC​AGC​AGC​ACA​AG	59.9	50	207	10
	3′R: GCG​ACA​CGA​GAA​ATG​AGT​GA	59.99	50		10
CteZFP66	5′F: TTT​GTG​GGA​GGA​AGT​TTT​GG	59.94	45	226	10
	3′R: CGC​CCT​CTT​TAA​CTC​GTC​TG	60.01	55		10
CteZFP85	5′F: AGT​CGG​ACC​GAT​ATG​TTT​GC	59.96	50	245	10
	3′R: CCC​ATT​GTT​TGT​GAT​TGC​TG	59.96	45		10
CteZFP88	5′F: GAC​CAG​CAT​CAC​CAT​TTC​CT	59.93	50	224	10
	3′R: TTC​CTC​CCA​TTG​TCG​AAG​AC	60.05	50		

### Three-dimensional protein structure and SSRs prediction

Three-dimensional (3D) structures of candidate *C. teeta* C2H2-zinc finger proteins were modeled on the basis of homology modeling using the SWISS-Model (Waterhouse et al., 2018). In addition, global model quality estimation (GMQE) was used as a measure of model quality (Waterhouse et al., 2018). GMQE scores were between 0 and 1, with higher scores indicating more reliable models (Wang et al., 2023). TBtools (Chen et al., 2020) was used to predict the candidate gene specific SSRs.

## Results

### Identification of the C2H2-zinc finger protein family in *C. teeta*

In order to comprehensively identify genes encoding members of the C2H2 transcription factor family in the genome of *C. teeta*. A total of one hundred candidate C2H2-zinc finger genes were identified (Supplementary Table S1). The number of amino acids (aa) of the predicted proteins encoded by the 100 genes ranged from 126 to 2229. In order to understand the possible function(s) of the proteins encoded by these genes, information regarding their physicochemical properties was essential. For instance, protein separation could be performed based on their molecular weight and isoelectric point characteristics (Tartaglione et al., 2016).

These analyses revealed that the isoelectric point of the predicted C2H2-zinc finger proteins ranged from 4.94 to 11.42. In addition, the relative molecular weight of the proteins varied significantly, ranging from 13934.75 to 250935.56 Da. The instability index, aliphatic index, and subcellular localization of the proteins were also predicted. The results showed that 94 were unstable, and only 6 were stable (Gasteiger et al., 2005). All the proteins were predicted to be hydrophilic with an aliphatic index ranging from 41.33 to 95.42. Most of them were predicted to be localized in the cell nucleus. However, six were predicted to be localized in the chloroplast, and another six were predicted to be co-localized in both the chloroplast and nucleus (Supplementary Table S3).

*C. teeta* C2H2-zinc finger genes with a tandem ZF domain in a single array or in more than one array were assigned accordingly to the A and B sets, while those containing a single ZF or dispersed ZFs were assigned to set C (Englbrecht et al., 2004; Arrey-Salas et al., 2021). These analyses revealed that 27 proteins containing tandem ZF arrays were assigned to set A, 3 proteins containing more than one ZF array were assigned to set B, and 70 proteins containing a single ZF or scattered ZFs were assigned to set C (Supplementary Figure S2; Supplementary Table S3).

### Phylogenetic tree and conserved motif analysis of *C. teeta* C2H2-zinc finger gene family

**FIGURE 1:**
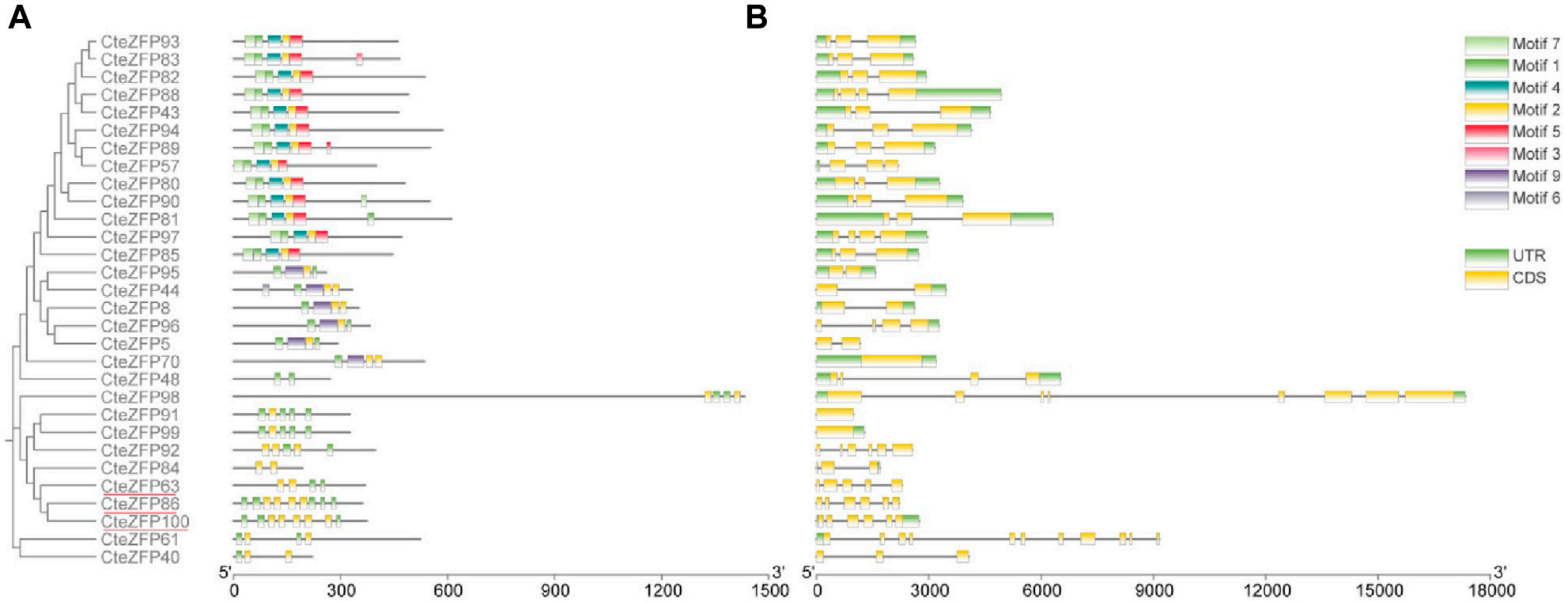
Phylogenetic relationship, gene structure and motif composition of *C. teeta* C2H2-zinc finger genes from A-set and B-set. **(A)** An unrooted phylogenetic tree was constructed from alignments of the amino acid sequences of A-set (Black font) and B-set (Red line). **(B)** Exon/intron structures of *C. teeta* C2H2-zinc finger genes.

In order to understand the phylogenetic relationships among the CteZFP sets in *C. teeta*, an unrooted phylogenetic tree was constructed, from alignments of the amino acid sequences of set A and set B (Figure 1A). The A-set and B-set CteZFP proteins were classified into two major groups (I and II) containing 20 and 10 predicted proteins respectively. In addition to CteZFP48, all the *C. teeta* C2H2-zinc finger proteins in group I contained motif 1 and motif 2. Twelve CteZFP proteins in group I contained motif 4, motif 5 and motif 7; six contained motif 9. Among the 10 CteZFP proteins in group II, all contained motif 1 and motif 2. Three proteins (CteZFP86, CteZFP100 and CteZFP63) belonged to the B-set, these proteins contained at least 2 copies of motif 1 and motif 2 (Figure 1B).

**FIGURE 2:**
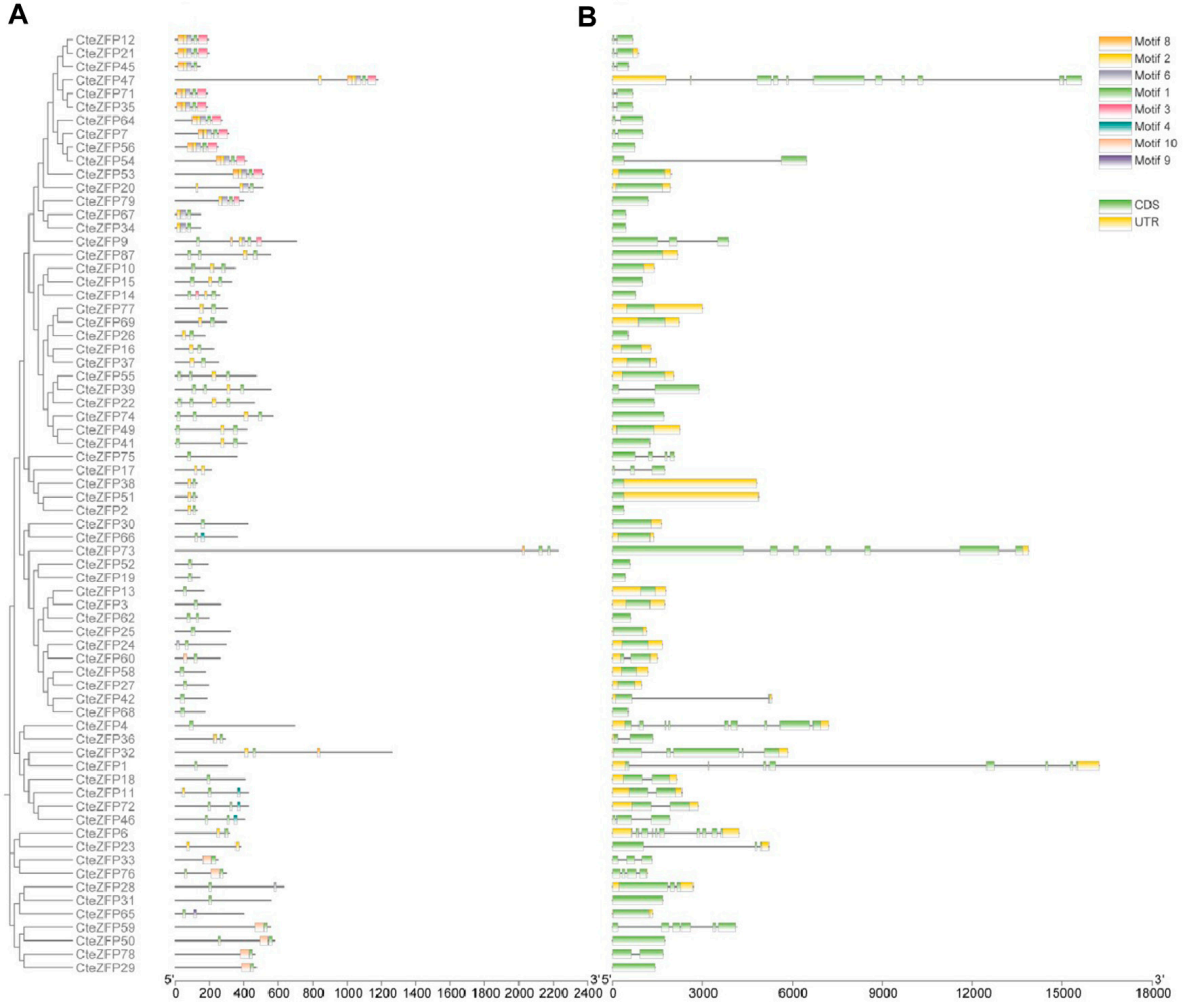
Phylogenetic relationships, gene structure and motif composition of *C. teeta* C2H2-zinc finger genes from C-set. **(A)** An unrooted phylogenetic tree was constructed from alignments of the amino acid sequences of C-set. **(B)** Exon/intron structures of *C. teeta* C2H2-zinc finger genes.

The CteZFP proteins of set C were further classified into seven major classes (I-VI) (Figure 2A), with 16, 14, 5, 15, 8, and 11 proteins in each group, respectively. Group I had no unique motifs. In group II, all proteins contained motif 1 and motif 2. In group III, all proteins contained motif 1 (except for CteZFP84). All proteins present in group IV, contained motif 1, one protein (CteZFP60) contained motif 10, one protein (CteZFP24) contained motif 6 and one protein (CteZFP73) contained motif 8. Another (CteZFP66) contained motif 4. All the proteins in group V contained motif 1. In summary, all proteins contained motif 1 or motif 2, but the number of motif 1 occurrences varied (Figure 2B). These variances in putative protein motifs further reveal the molecular diversification present in the CteZFP gene family.

Further analysis of gene structure within this family was conducted based upon the phylogenetic tree. The gene structure analysis revealed diversity within the A-set, ranging from intron-less genes, to genes containing ten introns (Figure 1C; Supplementary Table S4). These differences could be observed among different groups through phylogenetic analysis (Figure 1A). Genes without introns and those with more than one intron were also grouped together. However, the genes included in group I predominantly contained two introns in the A-set (Figure 1C; Supplementary Table S4). The genes included in the B-set contained between two to five introns (Figure 1C; Supplementary Table S4). In the C-set, 57.1% (40 genes in total) did not contain introns, 31.4% (22 genes) contained one or two introns, and 11.4% (8 genes) possessed between three to ten introns. The gene with the highest number of introns was CteZFP47 which was shown to contain ten introns (Figure 2C; Supplementary Table S4).

### Chromosomal locations of *C. teeta* C2H2-zinc finger genes

**FIGURE 3:**
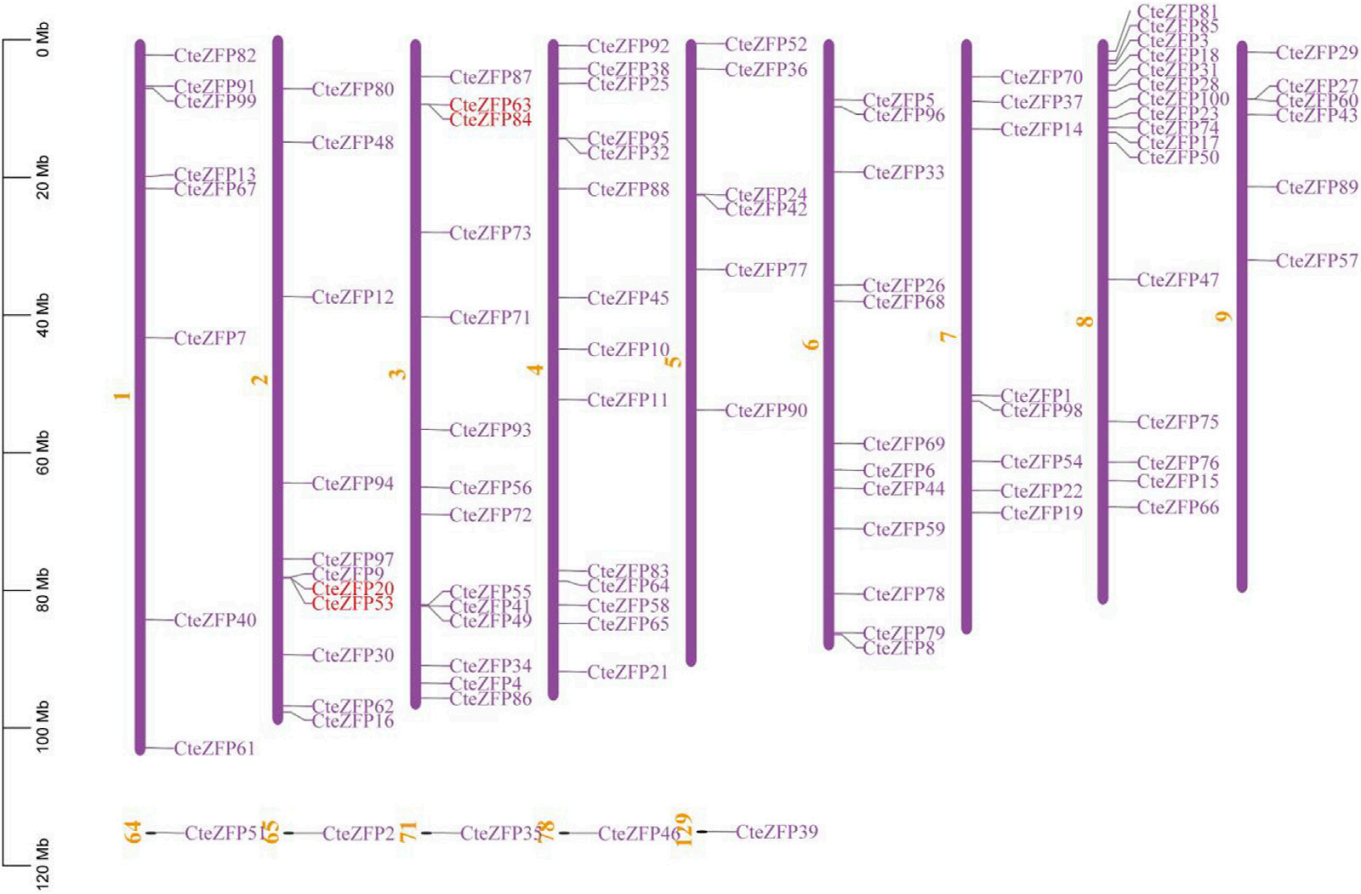
Genomic distribution of *C. teeta* C2H2-zinc finger genes. Five genes could not be mapped to any chromosome. Red lines represent the tandemly duplicated genes.

In order to study the distribution of C2H2-zinc finger genes on the chromosomes of *C. teeta*, chromosomal mapping analysis was conducted. Ninety-five C2H2-zinc finger genes of *C. teeta* were mapped across all 9 chromosomes, the remaining five genes could not be mapped to any chromosome. The chromosomal distribution of the *C. teeta* C2H2-zinc finger genes was not uniform. Chromosome 8 contained the highest number (16) of these genes, whilst chromosomes 5 and 6 contained the lowest number, each containing 6 C2H2-zinc finger genes (Figure 3).

**FIGURE 4:**
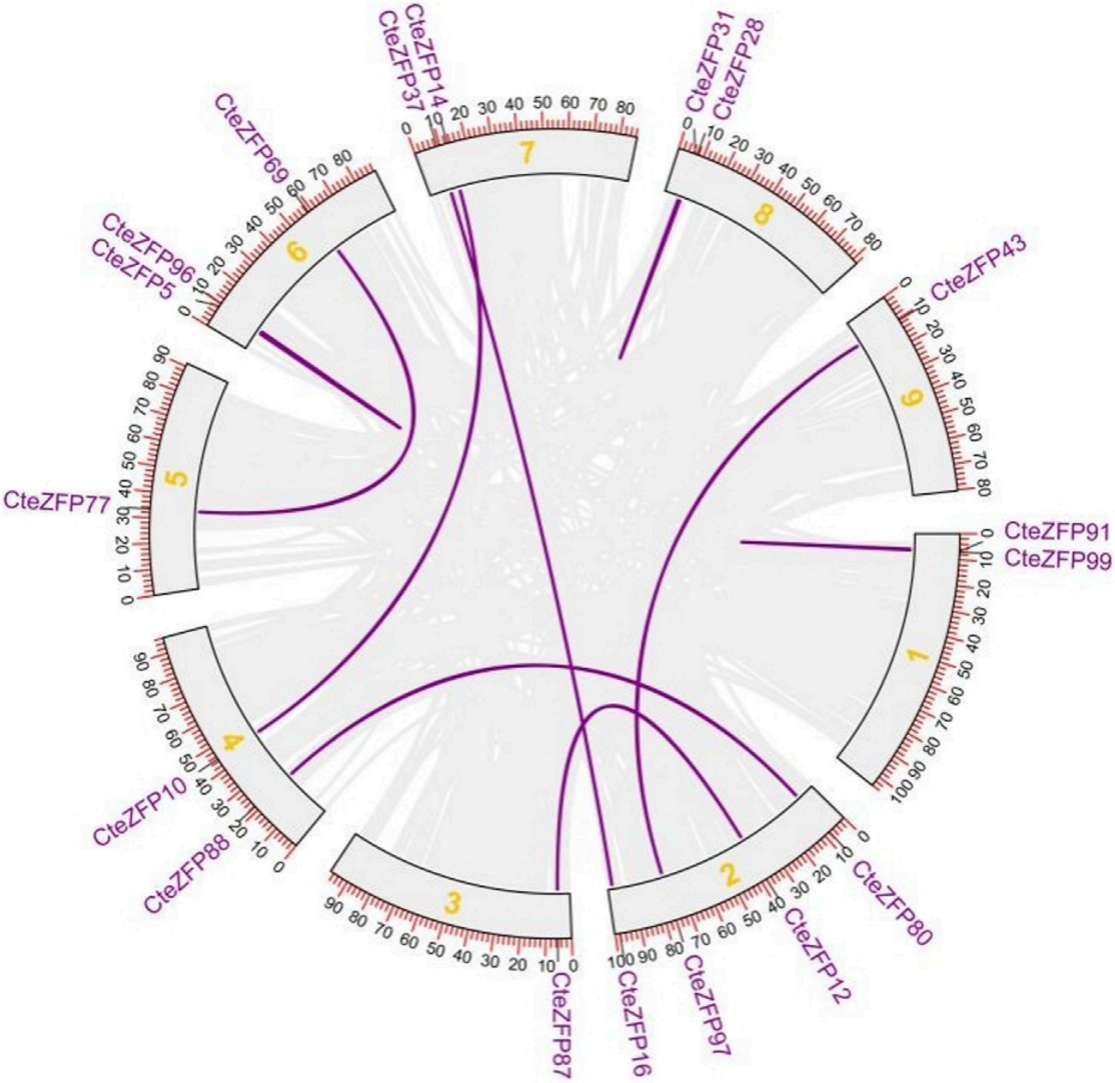
The segmental replication events of *C. teeta* C2H2-zinc fingergenes. Gray lines indicate all synteny blocks in the *C. teeta* genome and the purple lines indicate segmental duplication of *C. teeta* C2H2-zinc finger. The chromosome number is indicated at the middle of each chromosome.

Gene duplication events often lead to the expansion of gene families and the complexity of plant genomes resulting in an increase in the number of gene families present (Blanc and Wolfe, 2004; Salih et al., 2019; Lai et al., 2020). In order to explain the diversity within the C2H2-zinc finger transcription factor family of *C. teeta*, we investigated the segmental and tandem duplication events using TBtools software. A total of 2 pairs of tandem duplicated genes and 9 pairs of segmental duplicated genes were identified (Figures 3, 4).

To further investigate the evolutionary selective pressures, the nonsynonymous (Ka), synonymous (Ks) and Ka/Ks were estimated for pairs of tandem and segmentally duplicated C2H2 zinc finger genes. The results obtained revealed that all nine duplicated pairs of genes had Ka/Ks ratios lower than 1 (Table 2), indicating that these genes were undergoing purifying selection.

**TABLE 2:** Analysis of duplication events of C2H2-zinc finger genes pairs.

Gene 1	Gene 2	Ka	Ks	Ka/Ks		Negative selection
*CteZFP20*	*CteZFP53*	0.2930	0.4003	0.7320	tandem	Yes
*CteZFP63*	*CteZFP84*	0.2509	0.3684	0.6811	tandem	Yes
*CteZFP91*	*CteZFP99*	0.0026	0.0000	0.0000	segment	Yes
*CteZFP31*	*CteZFP28*	0.0382	0.0568	0.6724	segment	Yes
*CteZFP96*	*CteZFP5*	0.0000	0.0000	0.0000	segment	Yes
*CteZFP12*	*CteZFP87*	0.5528	1.6257	0.3400	segment	Yes
*CteZFP80*	*CteZFP88*	0.3036	1.0920	0.2780	segment	Yes
*CteZFP16*	*CteZFP37*	0.2688	2.0345	0.1321	segment	Yes
*CteZFP97*	*CteZFP43*	0.3220	1.2636	0.2548	segment	Yes
*CteZFP10*	*CteZFP14*	0.5717	2.3418	0.2441	segment	Yes
*CteZFP77*	*CteZFP69*	0.2514	1.1514	0.2184	segment	Yes

This italic values means that these two genes belong to tandem or segment, e.g., *CteZFP20* and *CteZFP53* belong to tandem.

### Promoter *Cis*- element analysis of *C. teeta* C2H2-zinc finger genes

To further investigate the function of the C2H2 zinc finger genes the cis-acting promoter regulatory elements were analyzed. Previous studies had shown that promoter cis-regulatory elements can be classified into five main functional groups: transcriptional, cell cycle, hormonal, abiotic or biotic stress, and developmental (Mi et al., 2021).

**FIGURE 5:**
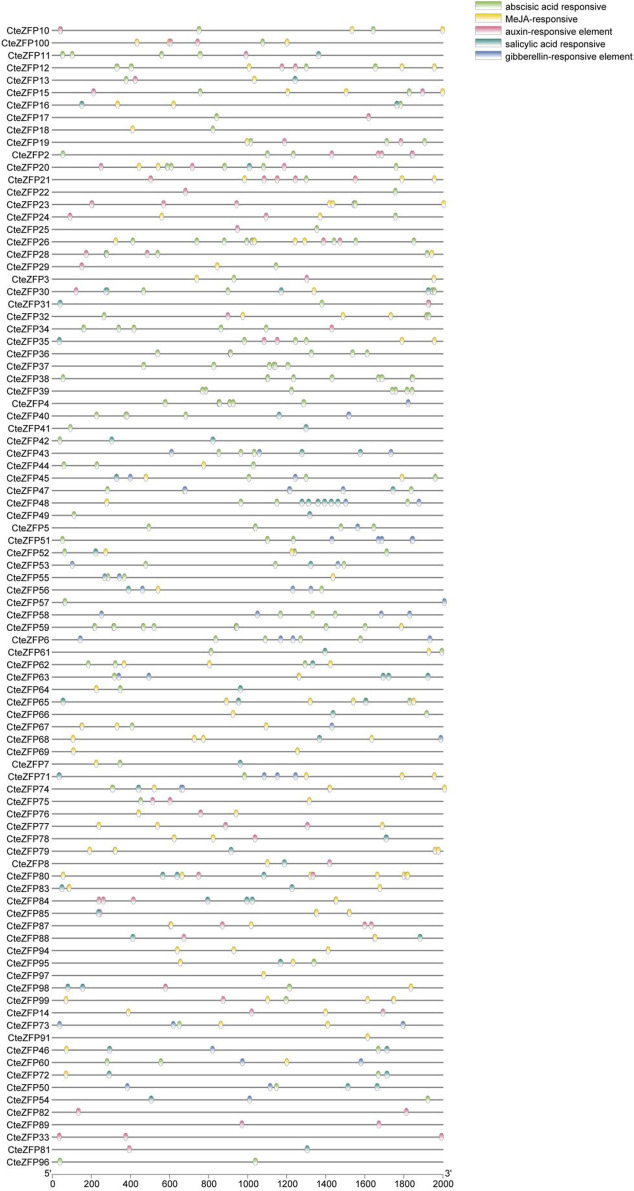
Distribution of various cis-acting elements in the promoter regions of *C. teeta* C2H2-zinc finger genes. The cis-elements of *C. teeta* C2H2-zinc finger genes were identified using the online PlantCARE website. Special cis-acting elements were selected (e.g., auxin-responsive elements), and the TBtools software was used to create the diagrams.

In the current study, it was found that 48.7% of the *cis*-acting regulatory elements, were related to transcription, 12.9% were associated with abiotic or biotic stress and 5.8% were associated with hormonal regulation (Figure 5; Supplementary Table S5).

Previous studies have shown that hormones play a key role in floral development. Thirteen ABA-responsive elements, 7 MeJA-responsive elements, 6 SA-responsive elements, 5 IAA-responsive elements, 5 GA-responsive elements, 3 ethylene-responsive elements were identified from our analyses. *CteZFP36, CteZFP37*, *CteZFP38,* and *CteZFP39* were found to contain ABA-responsive elements. *CteZFP82* and *CteZFP89* were found to contain IAA-responsive elements. It is of interest to note that, the promoter sequence of *CteZFP97* contained 13 ABA-responsive elements (Supplementary Figure S3), indicating a strong response of this gene to ABA signaling.

### Expression analyses of *C. teeta* C2H2 zinc finger genes in different floral organs

**FIGURE 6:**
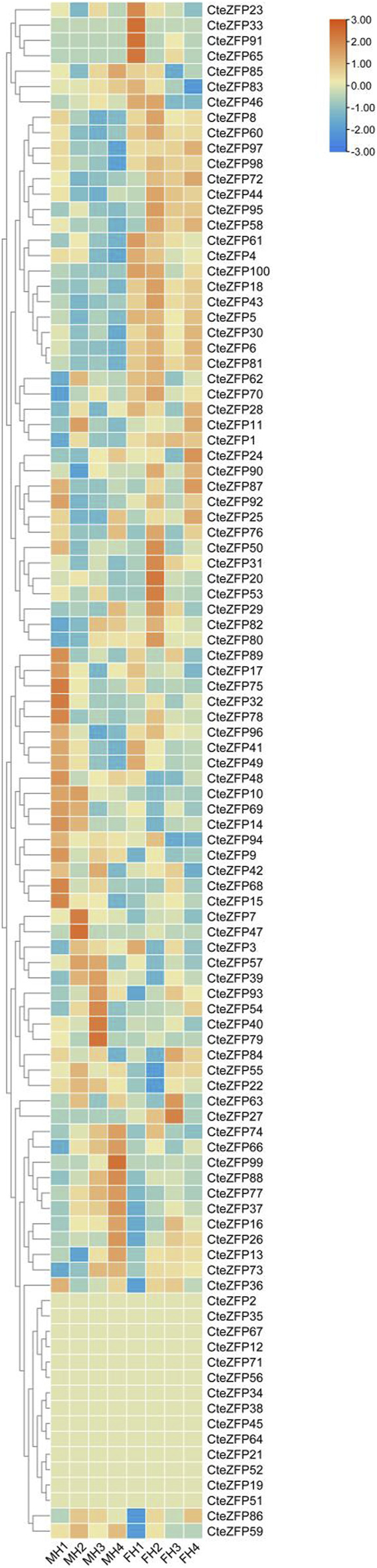
Heat map representation of *C. teeta* C2H2-zinc finger genes within two floral phenotypes at selected stages of flower development. MH (M-type): short pistil and long stamen phenotype; FH (F-type): long pistil and short stamen phenotype; 1 (stage 1): The bracts were dehiscent and florets unexposed; 2 (stage 2): The florets were just exposed, and sepals were unexpanded; 3 (stage 3): The sepals had fully expanded; 4 (stage 4): The anthers had dehisced and had begun to disperse pollen.

**FIGURE 7:**
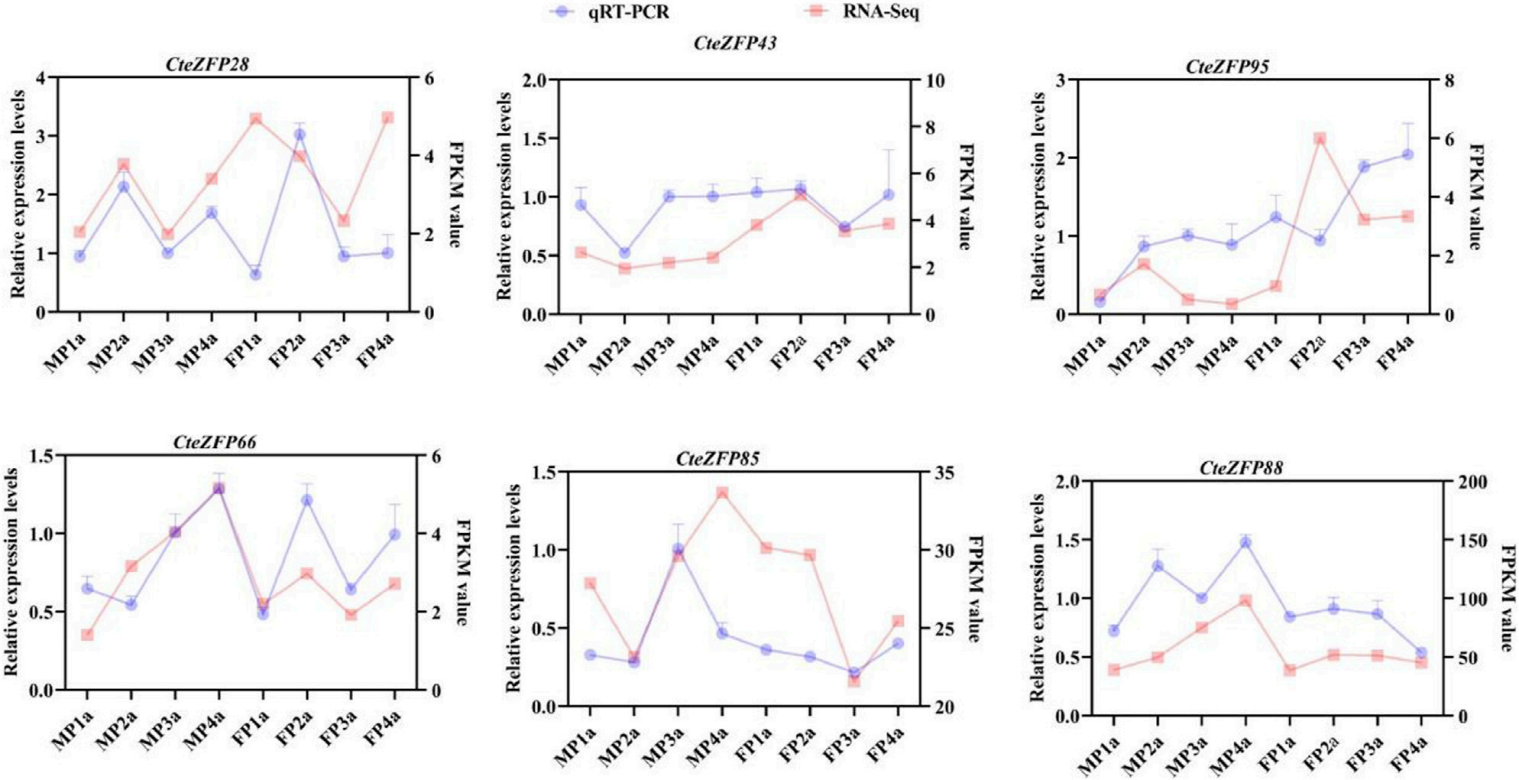
Expression profiles of selected *C. teeta* C2H2-zinc finger genes analysed by qRT-PCR analysis in different tissues.

In order to further investigate the role of the C2H2 zinc finger genes in floral organ development, the expression patterns of these genes were analyzed using RNA-Seq data. The differential expression pattern is shown in the heatmap figure (Figure 6). The expression levels were compared between the F and M phenotypes in flowers at different developmental stages. Among the C2H2 zinc finger genes studied, 13 showed little or no expression during floral development, in either flower type. Ten genes belonging to the A-set and B-set genes displayed differential patterns of expression between the M and F type flowers. Eight genes (*CteZFP90*, *CteZFP95*, *CteZFP43*, *CteZFP70*, *CteZFP44*, *CteZFP81*, *CteZFP80* and *CteZFP61*) were more highly expressed in F-type flowers and two genes (*CteZFP99* and *CteZFP88*) were more highly expressed in M-type flowers. In addition, a differential pattern of expression in 26 genes belonging to the C-set was found between the two flower types. Thirteen genes (*CteZFP10*, *CteZFP69*, *CteZFP14*, *CteZFP7*, *CteZFP21*, *CteZFP47*, *CteZFP54*, *CteZFP39*, *CteZFP79*, *CteZFP59*, *CteZFP66*, *CteZFP77* and *CteZFP37*) were more highly expressed in M-type flowers and 15 genes (*CteZFP28*, *CteZFP60*, *CteZFP18*, *CteZFP30*, *CteZFP6*, *CteZFP72*, *CteZFP58*, *CteZFP76*, *CteZFP31*, *CteZFP20*, *CteZFP46*, *CteZFP23*, *CteZFP65*, *CteZFP33* and *CteZFP4*) were more highly expressed in F-type flowers (Figure 6). In order to further analyze the expression patterns of these genes, qRT-PCR was used. The qRT-PCR results showed that the expression levels of *CteZFP28, CteZFP43* and *CteZFP95* in F type plants were higher than those in M type, and the expression levels of *CteZFP66*, *CteZFP85* and *CteZFP88* in M type plants were higher than those in F type. These data were consistent with the transcriptome data (Figure 7).

### Three-dimensional structure and SSRs prediction of *C. teeta* C2H2 zinc finger candidate genes

**FIGURE 8:**
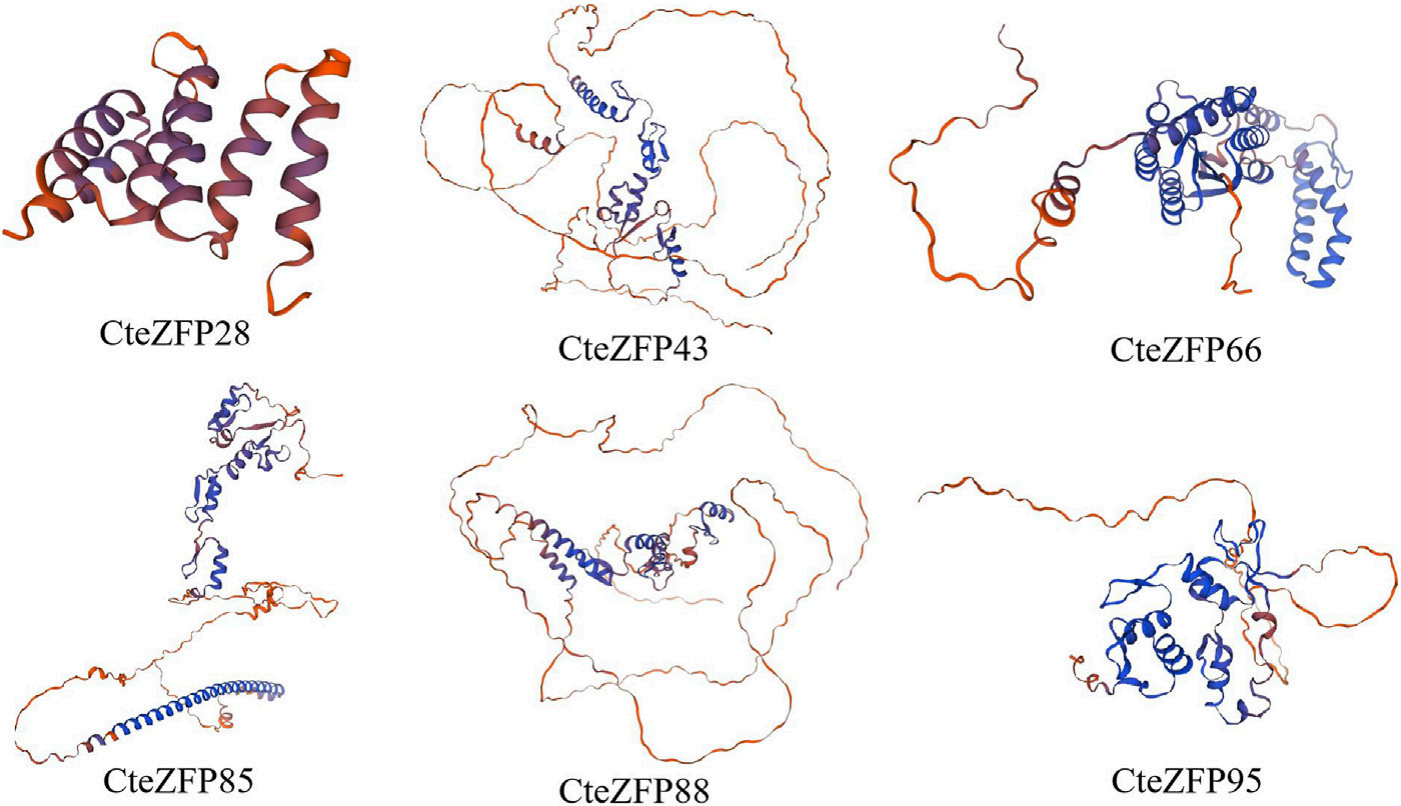
Three-dimensional (3D) protein structures of 6 C*. teeta* C2H2-zinc finger candidate protein.

We utilized SWISS-Model software to predict the 3D protein structures of 6 *C. teeta* C2H2 Zinc Finger Candidate Genes (Figure 8). In the six models, apart from CteZFP28, the similarity of the other five sequences is above 70%, with GMQE values ranging from 0.48 to 0.68 (Supplementary Table S6), which aligns with the widely accepted threshold for successful modeling (Xiang, 2006). All six models are monomeric and oligomeric, with CteZFP28 and CteZFP66 mainly composed of Alpha helices, while CteZFP43, CteZFP95, CteZFP85, and CteZFP88 are mainly composed of Random coils (Figure 8). Predict the candidate gene specific SSRs. (*CteZFP88*) SSR, repeating unit length 3, Number of repeating units 5 (Supplementary Table S7).

## Discussion

The C2H2-zinc finger protein family plays several important roles in plant growth and development, and has been extensively studied in various species including *Arabidopsis thaliana* (Englbrecht et al., 2004), *Zea mays* (Wei et al., 2016), *Triticum aestivum* (Li et al., 2021; Wu et al., 2022).

The subject of the current study, *C. teeta*, exhibits typical herkogamy phenomena during the specification of its floral organs. To investigate the potential role of C2H2-zinc finger proteins in floral organ formation, a genome-wide study of the zinc finger protein family was performed. One hundred putative C2H2-zinc finger protein genes were identified by whole-genome analysis. The physicochemical properties, gene structure, conservation motifs, promoter cis-elements, collinearity, gene duplications, and expression profiles of all one hundred genes and their predicted proteins were analyzed.

### Physicochemical properties and structure of *C. teeta* C2H2-ZFPs

The most fundamental and crucial characteristics of these functional proteins are their physicochemical properties (Sun et al., 2022). Our analyses of the predicted protein products of this gene family indicated that the C2H2-zinc finger proteins could be subdivided into different groups, each of which could fulfil a variety of physiological functions. Similarly, differences in gene structure and motif composition among gene subfamilies has been identified in studies of *Populus trichocarpa* as a possible cause of functional differences (Liu et al., 2015). Subcellular localisation analysis showed that the majority were predicted to localised in the nucleus, consistent with their role in binding to DNA (Takatsuji, 1999).

### Evolution of *C. teeta* C2H2-ZFP genes

The chromosome localization analysis revealed that the C2H2-zinc finger genes were located on 9 chromosomes with the exception of 5 genes that could not be localized. These genes were not evenly distributed. Such an uneven chromosomal distribution of ZFP genes has also been observed in other species such as maize (Wei et al., 2016), tomato (Shang et al., 2021) and wheat (Wu et al., 2022). Gene duplication events represent gene family expansion and genome complication in plants and often result in an increase in numbers of gene families (Cannon et al., 2004; Magadum et al., 2013). The current study identified a total of 2 pairs of tandemly duplicated genes, and 9 pairs of segmentally duplicated genes. Duplicated genes can create new functions and improve plants’ ability to adapt to the environment (Lee et al., 2008; Airoldi and Davies, 2012; Huo et al., 2021). Further analysis was subsequently conducted on the Ka/Ks values of these genes. This finding is consistent with a recent report regarding the evolution of cotton C2H2-zinc finger genes under negative selection (Salih et al., 2019). This suggests that these genes were primarily evolving under negative selection. A total of 2 pairs of tandem duplicated genes and 9 pairs of segmentally duplicated genes were identified. The segmental gene duplication could play a significant role in the evolution and diversification of this gene family in *C. teeta*. Previous research found that the maize ZFP gene family was probably extended by tandem duplication and segmental duplication (Wei et al., 2016).

### Tissue specific expression and putative role in flower development

Transcriptome analysis revealed that 13 genes of the 100 C*. teeta C2H2-ZFPs* were not expressed during flower development. However, several genes were identified that showed high expression in specific floral tissues (*CteZFP95, CteZFP88, CteZFP66* et al.).

To further understand the functionality of these genes, we conducted a comparison with genes present in the *Arabidopsis thaliana* database and found that *CteZFP95* showed high homology to *AT1G34790 (AtWIP1/TT1)*. Previous studies have shown that the expression of AtWIP1/TT1 in carpel primordia, leads to the development of male flowers (Roldan et al., 2020). Further analysis of the promoter region of this gene revealed the presence of response elements for ABA, MeJA, and SA. Jasmonates (JA), including methyl jasmonate (MeJA). These response elements had the ability to influence cell expansion (Noir et al., 2013; Wasternack and Hause, 2013; Kazan, 2015) and played a crucial regulatory role in plant development and defense processes (Yuan and Zhang, 2015). Influencing the transduction of JA signals can affect the pistil of tomatoes, causing the pistil to be longer than the stamens. Therefore, the homologous gene *CteZFP95* may fulfil a similar role in the formation of herkogamy in *C. teeta*. However, confirmation of this hypothesis will require further experimentation.

## Conclusion

In conclusion, we have identified one hundred putative C2H2-zinc finger genes to be present in the *C. teeta* genome, that encode for proteins with at least one ZF conserved motif. These data allowed us to infer an intricate evolution history for this gene family due to the high diversity shown to be present in the proteins encoded by these genes. The detailed features of this gene family and its predicted protein products were also investigated, including the physicochemical properties, subcellular localization, phylogenetic relationships, exon-intron structures, conserved motifs, chromosomal locations, gene duplication events, cis-regulatory elements, gene expression patterns and qRT-PCR analysis. The results of this study provide the basis for future functional studies of the *CteZFP* gene family, and their detailed role(s) in floral development in this species.

## Data Availability

The original contributions presented in the study are included in the article/Supplementary Material, further inquiries can be directed to the corresponding author. All sequencing data were deposited at the NCBI Sequence Read Archive (SRA) (accession number: PRJNA973818) Transcriptome Analysis of Flower Development in Coptis teeta Wall. at https://dataview.ncbi.nlm.nih.gov/object/PRJNA973818?reviewer=lk2sb8em203d3o8vci36s6ilf2.

## Supplementary material

SUPPLEMENTARY FIGURE S1 The four selected stages of flower development are shown in plants exhibiting the M and F type floral phenotypes. Stage 1 (P1). The bracts were dehiscent and florets unexposed; stage 2 (P2): The florets were just exposed, and sepals were unexpanded; stage 3 (P3): The sepals had fully expanded; stage 4 (P4): The anthers had dehisced and had begun to disperse pollen.

SUPPLEMENTARY FIGURE S2 Phylogenetic trees of *C. teeta* C2H2-zinc finger proteins. Phylogenetic tree of C2H2-zinc finger proteins between *C. teeta* (circle, lightblue) and Arabidopsis (star, pink). The three set of the C2H2-zinc finger proteins are represented by different colors, A set (violet) (lightgreen), B set (lightblue), C set (lightgreen).

SUPPLEMENTARY FIGURE S3 Analysis of *Cis*-acting elements of *C. teeta* C2H2-zinc finger genes family.

SUPPLEMENTARY TABLE S1 Location, gene name and protein domains of C2H2-zinc finger genes identified in the *C. teeta* genome.

SUPPLEMENTARY TABLE S2 Detailed information regarding *C. teeta* C2H2-zinc finger gene family.

SUPPLEMENTARY TABLE S3 The classification criteria used according to ZF domain distribution.

SUPPLEMENTARY TABLE S4 Exon and intron distribution in the *C. teeta* C2H2-zinc finger gene family.

SUPPLEMENTARY TABLE S5 Distribution of various *cis*-acting elements in the promoter regions of *C. teeta* C2H2-zinc finger genes.

SUPPLEMENTARY TABLE S6 The detailed information of the predicted three-dimensional (3D) proteins.

SUPPLEMENTARY TABLE S7 Predict the candidate gene-specific SSRs.
