# Supplementary figures and images for: Genome-wide identification and expression analysis of the C2H2-zinc finger transcription factor gene family and screening of candidate genes involved in floral development in Coptis teeta Wall. (Ranunculaceae)
Source: Front Genet. 2024 Jan 22;15:1349673. doi: 10.3389/fgene.2024.1349673 (PMC10839097; doi:10.3389/fgene.2024.1349673)

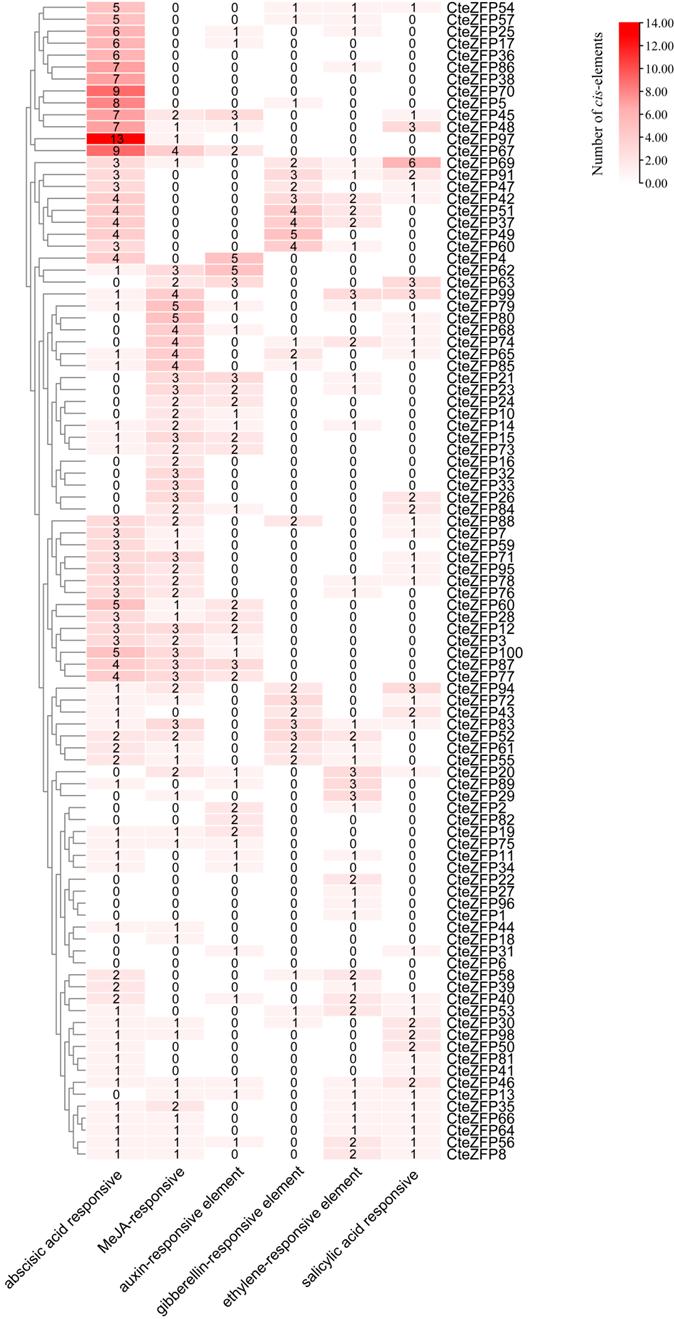

Supplement: Supplementary file 3 [file Image3.jpg]

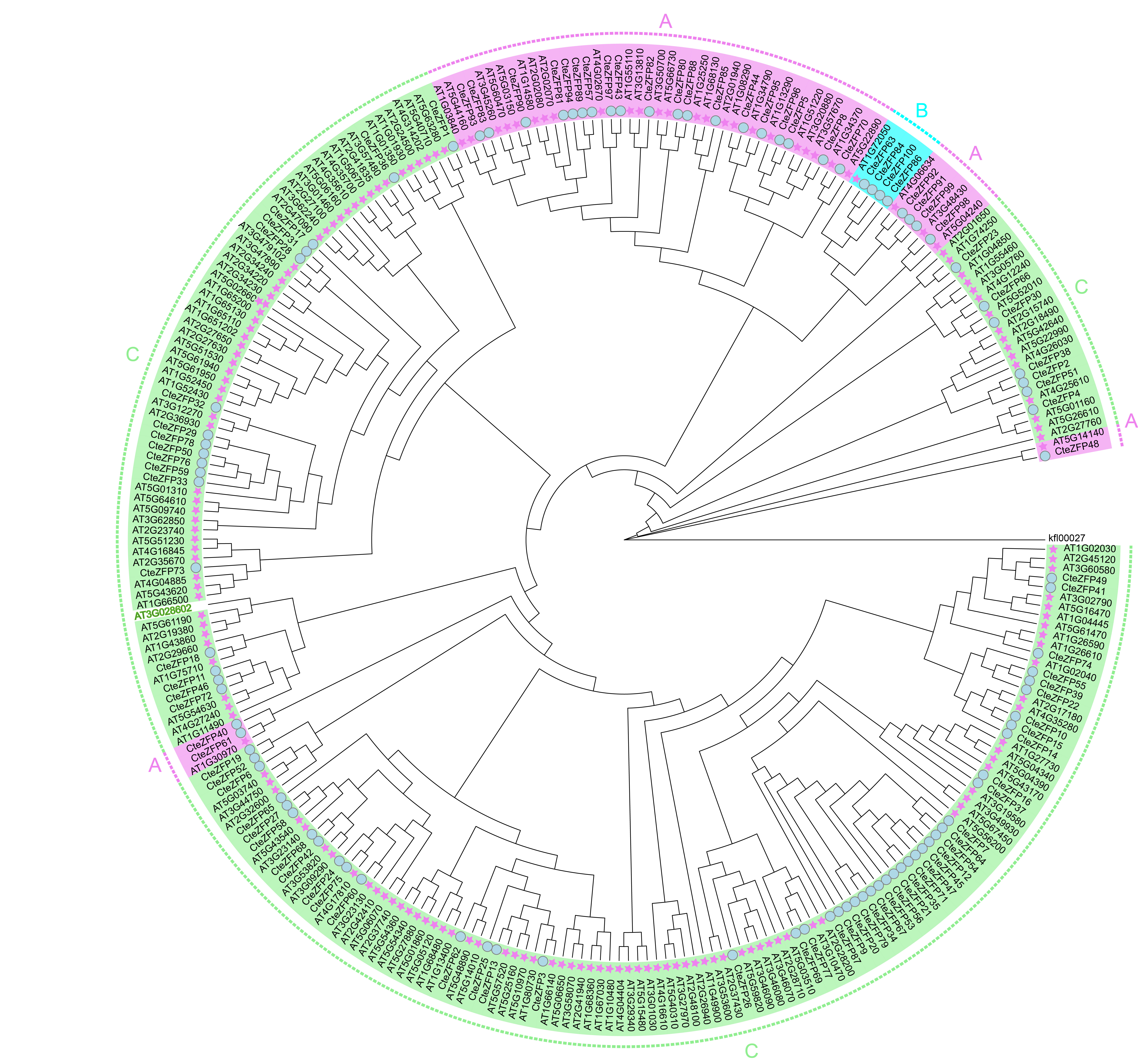

Supplement: Supplementary file 4 [file Image2.jpg]

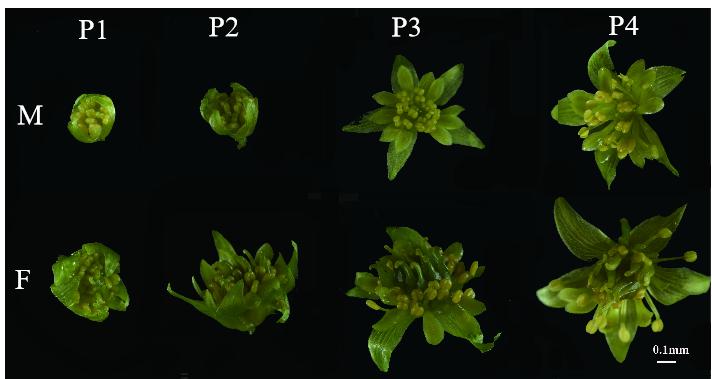

Supplement: Supplementary file 11 [file Image1.jpg]
